# A simple approach of applying blended learning to problem-based learning is feasible, accepted and does not affect evaluation and exam results—a just pre-pandemic randomised controlled mixed-method study

**DOI:** 10.1007/s00210-022-02306-3

**Published:** 2022-10-20

**Authors:** Ulrike Servos, Birger Reiß, Christoph Stosch, Yassin Karay, Jan Matthes

**Affiliations:** 1 Center of Pharmacology, Medical Faculty, University of Cologne, Cologne, Germany; 2 Student Dean’s Office, Medical Faculty, University of Cologne, Cologne, Germany

**Keywords:** Medical education, Learning, problem based, Blended learning, Method, teaching

## Abstract

We tested for feasibility, acceptance, and “non-inferiority” of small-group teaching applying blended learning (i.e., the integration of face-to-face and online instruction) to problem-based learning (bPbL) compared to conventional PbL (cPbL). In a just pre-pandemic, randomised controlled trial, 317 students attended either bPbL or cPbL groups. The first meeting of the bPbL groups took place online via written internet chat, while cPbL groups met on site. All groups met on site the second time. All students had the opportunity to attend lectures either on site or as videos on demand. We analysed student evaluation data, results in a final summative exam, attendance of lectures on site and use of lecture videos. Furthermore, we performed a qualitative analysis of student statements made in semi-structured group interviews about pros and cons of the bPbL approach. There was no difference between students of either bPbL or cPbL groups with respect to exam results (score: 14.3 ± 2.8 vs. 13.8 ± 2.7) or course evaluation. However, relatively more bPbL than cPbL students reported having used lecture videos, while the proportion of those attending lectures on-site was higher among cPbL students. Interviews revealed that some of the bPbL students’ experiences were unexpected and feared disadvantages seemed to be less severe than expected. Participation in a blended PbL format did not worsen course evaluations or exam results, but seemed to influence lecture attendance. The combination of face-to-face and digital elements could be suitable as a hybrid approach to digital instruction in the post-pandemic era.

## Introduction

The COVID-19 pandemic has impacted university teaching worldwide. Within a very short time, universities had to convert their face-to-face courses to digital formats. Meanwhile, many universities are returning to face-to-face teaching, which inevitably raises the question of whether and to what extent to return to conventional formats as well. Several surveys show that students would like to see a combination of face-to-face teaching and digital approaches, such as blended learning (Amir et al. 2020; Ma and Lee 2021). Blended learning can be defined as the integration of “face-to-face and online instruction” (Graham 2018). Given this definition, the variations of blended learning are manifold and very heterogeneous. In contrast, the core concept and process of PbL is quite well-defined (Davis and Harden 1999; Taylor and Miflin 2008). Even before the COVID-19 pandemic, we investigated the realisation of problem-based learning (PbL) by means of blended learning. We wondered whether and how parts of the PbL process could be moved from the seminar room to the internet. In a recent review and meta-analysis on digital PbL in health professions, the authors concluded that there is a need for more research on blended PbL (bPbL) in terms of digital approaches that enable partially distance-based PbL (Tudor Car et al. 2019). Of note, what is often referred to as blended PbL is rather a digitally supplemented conventional, i.e. on site PbL where information (e.g. the PbL case itself or additional findings like X-rays) are provided digitally (Moeller et al. 2010; Tudor Car et al. 2019).

Our main intervention was conducting the first PbL session as a written Internet chat instead of an on-site meeting, i.e. only the communication channel was different. Our study therefore aimed at collaborative learning in the broader sense of a non-inferiority study compared to conventional PbL (cPbL), which throughout takes place on site. We analysed student evaluation data, results in a final summative single-choice exam, as well as attendance of lectures on site and use of lecture videos. Furthermore, we performed a qualitative analysis of student statements made in semi-structured group interviews about pros and cons of the blended PbL approach.

## Methods

### Setting

**Fig. 1:**
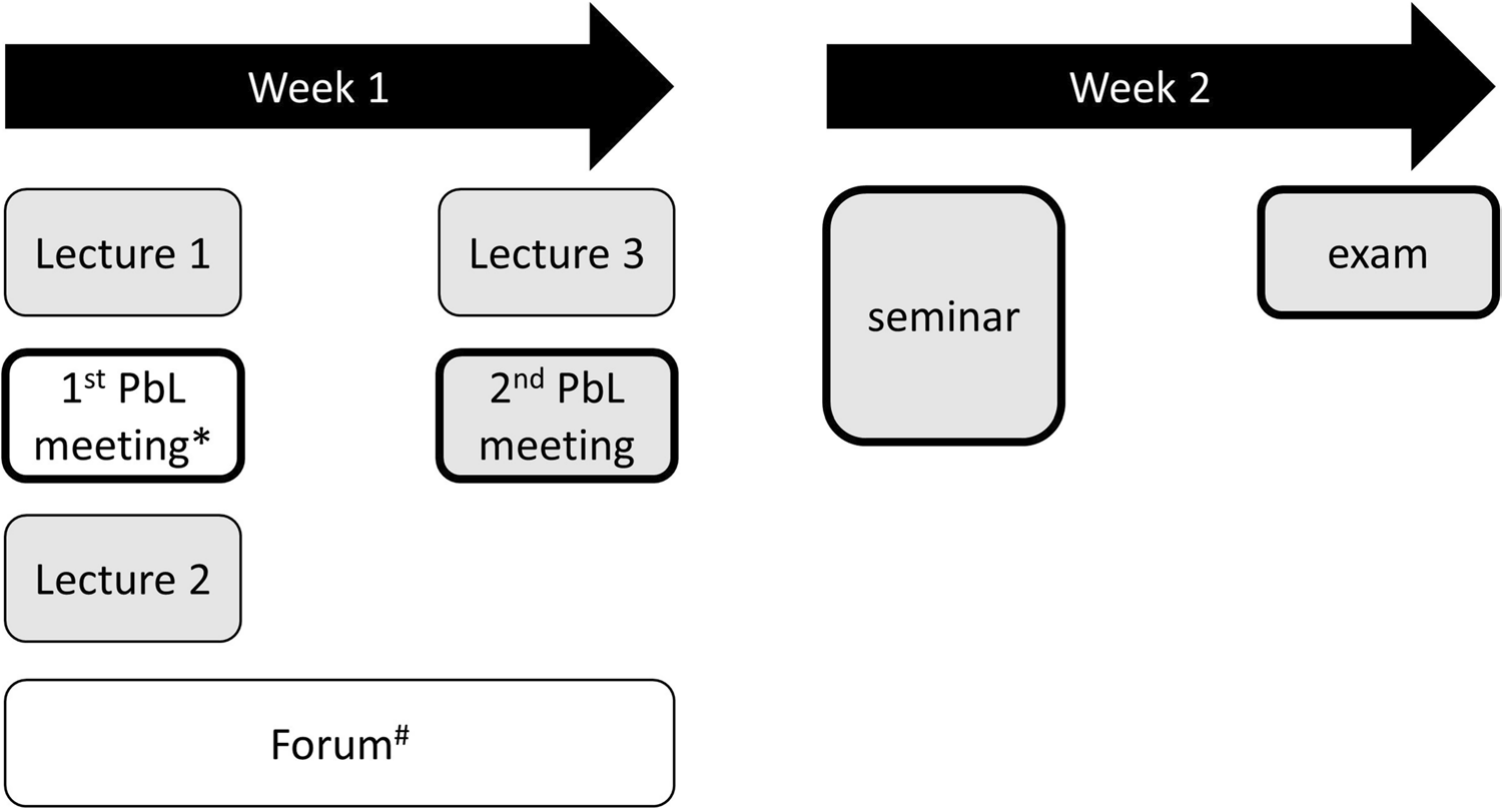
Course sequence and differences due to the intervention. Events in grey shaded boxes were the same for all students. Participation in events in bold framed boxes was compulsory. *First PbL meeting as a written Internet chat (bPbL groups) or on site (cPbL groups). ^#^Possibility of interaction via the internet platform ILIAS only for bPbL groups

The (pre-pandemic) setting of our study was a course dealing with the diagnosis of and treatment options for metabolic syndrome. This interdisciplinary course is a so-called competence area (literal translation of “Kompetenzfeld”), a format developed as element of the Cologne medical model curriculum (Zims et al. 2019) and offered by our Centre of Pharmacology for medical students in their third year of study. The course consists of three 1-h lectures, a 2-h seminar (including communication exercises) and small group teaching in a PbL format (Fig. 1). The course concludes with a separate summative written examination consisting of 20 single-choice questions on pharmacological and non-pharmacological aspects around metabolic syndrome. The exam was computer-based but written on site at the campus. Students knew PbL from a stand-alone 6-week course on basic pharmacology they attended in the preceding first half of the semester. PbL groups consisted of about 10 students who met twice for 1 h each with an interval of 2 days. A paper case describing a patient who needs to be treated for disorders that define metabolic syndrome was worked on under the guidance of a staff tutor according to the “Maastricht seven-jump approach” (Davis and Harden 1999). The course took place in four consecutive 2-week blocks, with about a quarter of the students in each block attending. In the current study, lectures were delivered in the lecture hall, but recorded during the first block and made available to all students, regardless of the block in which they took the course or of the type of PbL they attended, as a videos on demand uploaded to the ILIAS platform.

### Intervention

It is noteworthy that all students had recently attended another 6-week PbL course on site and were therefore familiar with PbL. In the blended PbL (bPbL) of our study, the first meeting of the PbL groups did not take place on site as usual, but the live communication took place exclusively via a written online chat on the ILIAS learning platform (www.ilias.uni-koeln.de). Of note, the chat feature provided by ILIAS at the time of the study was quite basic. The posts were only displayed in order of appearance and there was no possibility to reply directly to a particular post. It was not possible to send files (e.g. PDF or audio files) via the chat. Like the students, the tutor could only communicate via chat. The PbL case was provided separately as a PDF as well via ILIAS. The students had to read the PbL case themselves, as it could not be displayed via the chat. Conventional PbL (cPbL) groups met on site in seminar rooms as usual. All groups were asked to work on the PbL case up to the formulation of their own learning objectives in this first meeting. The second meeting, in which the research results are discussed together, took place for all groups on site. The only other difference between bPbL and cPbL groups was that the former had the opportunity to exchange information during the research phase between the two meetings via a forum that was also provided via ILIAS.

### Study design

In a pilot phase (winter term 2017/2018), students were asked to voluntarily participate in bPbL groups. Nineteen students from two bPbL groups and 29 students from three cPbL attended the pilot study, i.e. they filled in the questionnaires described below. The pilot phase was to check for technical feasibility of our approach, to validate the questionnaire (see below), and to check for feasibility of our guideline for semi-structured interviews. The main study was a randomised controlled trial. Thus, in the main study phase, 317 students were randomly allocated to 12 bPbL and 27 cPbL groups, respectively. Random numbers generated in Excel decided which PbL group the students participated in and thus whether this was a cPbL or a bPbL group. Since the study was also intended to investigate the feasibility of this blended PbL approach, and since it was unclear whether the students might be at a (perceived) disadvantage, we decided to use fewer bPbL groups than cPbL groups. While attendance of the PbL meetings was mandatory, filling in the questionnaire was voluntary. The final written exam was a summative exam, so most students took it at the end of the course.

### Questionnaires

At the time of the final exam, students were asked to fill in paper-based questionnaires. These questionnaires referred to students’ attitudes towards computer-based learning (CbL), their familiarity with the ILIAS platform, satisfaction with the course (including work of the PbL group, satisfaction with the tutor, perceived exam preparation), lecture attendance (either on site or by watching the videos), and the learning time spent apart of the PbL meetings. Items on CbL and tutor qualification were taken from questionnaires used in previous studies (Matthes et al. 2002, 2008; Hahne et al. 2005). Some questions were put only to students either attending a bPbL or a cPbL group, respectively (e.g. “I don’t think that the use of computer-based learning systems would be a gain for me” to cPbL and “When chatting in ILIAS, I found the collaboration within my PbL group to be productive and efficient overall” as well as “In the forum, I would have liked to see more participation from the group members overall” to bPbL attendees only). We used Likert scales from 1 (= true) to 5 (= not true). Regarding lecture attendance, options to choose from were “all”, “some” and “none”. Learning time was to be indicated by choosing “ < 1 h”, “1–2 h”, “2–3 h”, “3–4 h” or “ > 4 h”. Factor analysis using varimax rotation revealed three scales (defined by factor loadings ≥ 0.5 (Cleff 2015)) that we called “sceptical about CbL” (6 items), “satisfaction with the course” (4 items) and “ILIAS familiarity” (2 items), respectively.

### Interviews

Semi-structured group interviews were conducted with 7 bPbL and 7 cPbL groups. Entire groups were each interviewed by their PbL tutor (*n* = 5) or by the first author. Pre-formulated questions put to prompt feedback aimed at advantages and disadvantages of either bPbL or cPbL. Interviews were audio taped and subsequently transcribed. According to Mayring, an inductive qualitative content analysis was performed (Mayring 2010). Categories were formed by the first author based on the issues raised and then differentiated into pro and con arguments in regard to bPbL. The definitions of the categories and the assignment of quotations and categories were independently verified by two other persons. Although there were more cPbL than bPbL groups overall, we selected equal numbers for the interviews to limit the number of students who were additionally involved, to limit the effort required for data acquisition and analysis and to facilitate semi-quantitative comparison of the data.

### Statistics and ethical issues

With our quantitative analysis, we tested the hypothesis that there are differences between cPbL and bPbL groups in evaluation, exam or lecture attendance to imply non-inferiority if the initial hypothesis were to be rejected. For comparison of exam results and evaluation items, a Mann–Whitney test was applied. Proportion of lecture attendance or use of videos was compared by 2 × 2 contingency tables and Fisher’s exact test. Throughout, *p* < 0.05 was considered as indicating statistically significant differences. The local ethics committee gave the study a favourable evaluation (ID: 18–106).

## Results

### Evaluation results

**Fig. 2:**
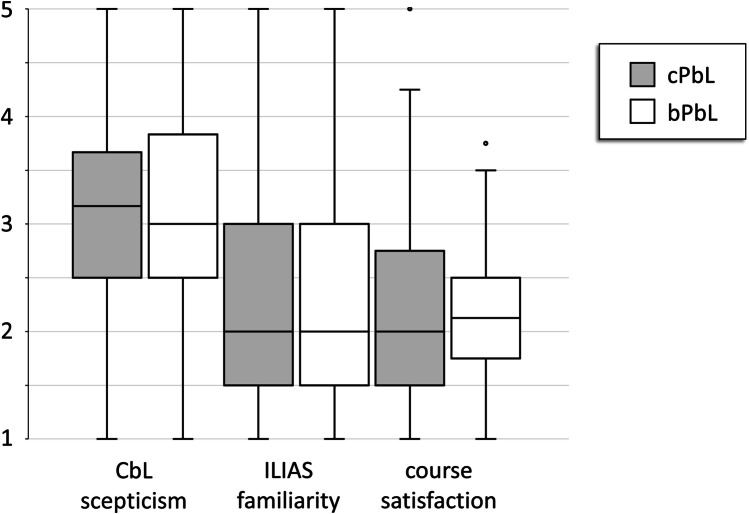
Evaluation data of the scales “sceptical about computer-based learning” (CbL), “familiar with the online platform ILIAS” and “satisfied with the course”. For every item, a Likert scale from 1 (= true) to 5 (= not true) was used. Items underlying a respective scale were averaged. Resulting scores from students attending conventional or blended problem-based learning (cPbL or bPbL) are shown as boxplots depicting median, 25th and 75th percentiles. *n* = 179–188 (cPbL) and *n* = 75–78 (bPbL), respectively

During the pilot phase, students voluntarily attending a bPbL group appeared to be significantly less sceptical about CbL compared to students who did not choose the bPbL approach (not shown). In the randomised main study, however, we found no difference between students having attended bPbL or cPbL groups with respect to the evaluation of CbL scepticism, familiarity with the Internet platform ILIAS, and satisfaction with the course (Fig. 2).

### Attendance of lectures on site and use of videotaped lectures

**Fig. 3:**
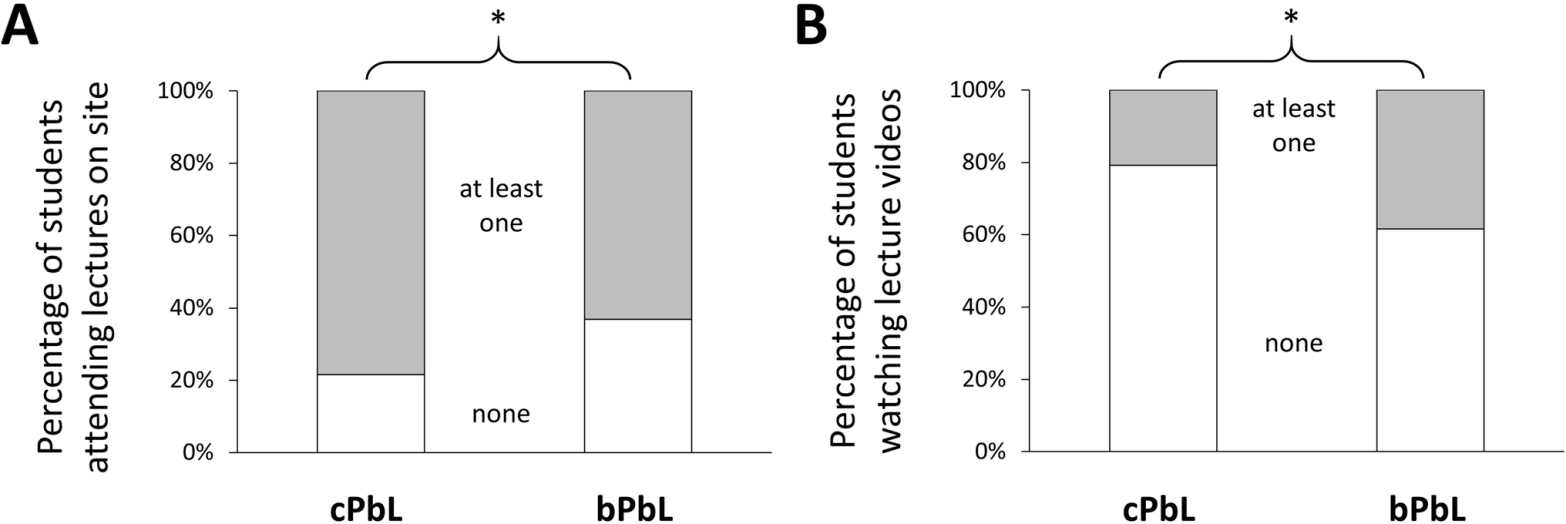
Percentage of students having attended at least one lecture on site (**A**) or watched at least one lecture video (**B**). cPbL conventional problem-based learning, bPbL blended problem-based learning. *p < 0.05 in a Fisher’s exact test

While 120 out of 153 (78%) cPbL students reported having attended at least one lecture on site, only 41 out of 65 (63%) bPbL students did (*p* < 0.05) (Fig. 3). Forty-four (29%) cPbL but only 10 (15%) bPbL students attended every lecture on site. In contrast, 21% of the cPbL students reported watching at least one lecture video, while 38% of the bPbL students did (*p* < 0.05). Nine percent of the cPbL but 18% of the bPbL students watched every video. Twelve percent of the cPbL students and 19% of the bPbL students stated to have neither attended a lecture on site nor watched a lecture video (*p* = 0.7).

### Exam preparation and results

**Fig. 4:**
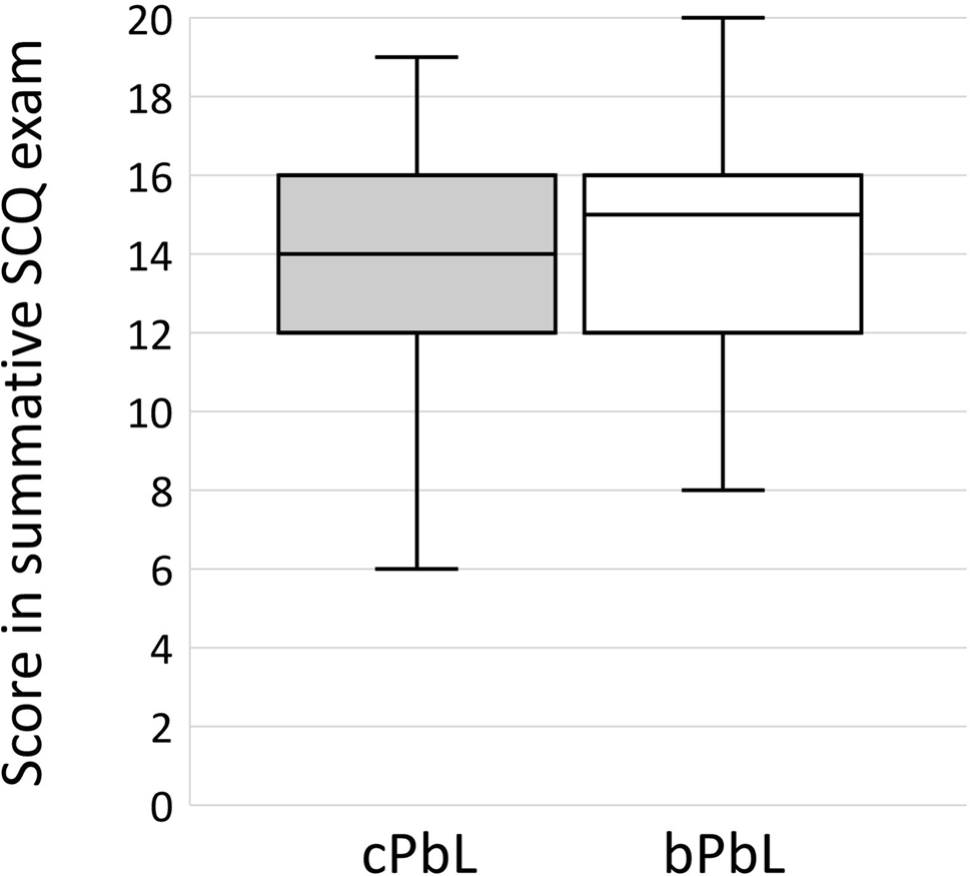
Results in a summative exam consisting of 20 single-choice questions (SCQ). Boxplots depict median, 25th and 75th percentiles. cPbL conventional problem-based learning (*n* = 204), bPbL blended problem-based learning (*n* = 92)

The amount of time students spent on the course in addition to PbL group sessions was similar (median of 3, i.e. 2–3 h in both cPbL and bPbL groups). With respect to the results in the final summative exam, there also was no difference between students attending a bPbL or cPbL group, respectively (Fig. 4).

### Results obtained from interviews

Categories derived from interviews with cPbL and bPbL groups can be taken from Table 1. Although the same number of bPbL and cPbL groups was interviewed, the qualitative content analysis yielded more discriminable statements from bPbL compared to cPbL groups (*n* = 169 vs. *n* = 134). In line with this, however, the mean interview duration with bPbL groups was also longer (7′ 41″ vs. 5′ 48″). The total number of categories covering students’ statements was similar for cPbL and bPbL groups (*n* = 38 and *n* = 43). Also, the proportion of individual statements supporting bPbL was similar (31% and 28%). In terms of pro but not con arguments, however, there were more categories addressed only by bPbL students (*n* = 8) than categories addressed only by cPbL students (*n* = 2).

**Table 1:** Main categories and sub-categories derived by content analysis from interviews with student groups (*N* = 7 each) attending a conventional (cPbL) or blended (bPbL) format of problem-based learning. (Sub-) Categories were further divided in those supporting (pro) and those being sceptical about the bPbL approach (contra). Frequency of statements corresponding to a respective (sub-) category are given as absolute numbers

Main category	Sub-category	Explanatory notes on (sub-) categories	Pro bPbL		Contra bPbL	
			By bPbL	By cPbL	By bPbL	By cPbL
Activity	General	Concerning the participation of students	3	2	2	7
Anonymity	General	Unknown counterpart, as no picture and abbreviation instead of plain name			2	2
Atmosphere	General	Learning climate	4	2		
Effort	General	Effort required	1		2	
	Journey	Reach a venue	7	11		
	Typing	Write on the keyboard			6	4
	Time	Time expenditure versus time saving	2	5	11	4
Eye contact	General	Only when face to face				2
Data privacy	General	chat for participants also visible afterwards			2	
Documentation	General	the chat history as documentation	1	2		
Doubling	General	Overlapping content of posts typed at the same time			10	1
Effect	General	Effects of the meeting			1	
	Learning	Learning effect in particular	1		11	8
Efficacy	General	Effect per time			4	
	Time	Delay due to typing			2	
Simple	General	Easy to implement				1
Flexibility	General	Not further specified	1	5		
	Place	regarding choice of place		2		
	Time	Regarding saved time	1		1	
Content	General	Content-related deficits			2	3
Communication	General	Easier on site				3
	Effort	costs more effort in the chat			1	
	Performance	Communication forms lacking, linguistic reduction			27	15
	Content	Better understanding as a result of direct communication				2
	Body language	Missing in chat				1
	Depth	simultaneous typing and/or linguistic reduction hampers in-depth discussion			4	5
	Typing	Restricts communication				1
Coordination	General	complicated by simultaneous activities				1
Concentration	General	Enhanced vs. decreased	4		1	
Learning format	General	Suitable vs. unsuitable	1		1	4
Postprocessing	General	Possible immediately after the meeting, if need be using the chat history	1	1		
Traceability	General	Writing promotes understanding, disorganised posts make it difficult to follow the discourse	2	1		1
Organisation	General	More confusing			3	
Choice of place	General	Promotes independence	10	5		
Face-to-face contact	General	f2f fosters social contacts and direct interaction			6	13
Convenience	General	Web-based is more convenient	3	1		
Productiveness	General	Talk is more productive than chat				1
Search	General	Access to the internet is an advantage vs. reduces the need to retrieve one’s own knowledge	2		2	1
Speed	General	Faster workflow on site				2
Social aspects	General	e.g. face-to-face strengthens social competence			3	1
Structure	General	Chat is not well-arranged			3	4
Technique	General	Easy to handle vs. depending on e.g. quality of web access	6		9	7
	Digitalisation	Web-based approach corresponds to the general development		1		
Visualisation	General	on-site notes are visible to all (e.g. on the blackboard)				2
Preparation	Professional life	Preparation for the (digital) working environment	1			
	Seminar	PbL case can be read unhurriedly	1			
Purposeful	General	Not purposeful			1	
No. of (sub-) categories addressed			19	13	23	26
No. of mentions in total			52	39	117	96

In the following, “C “ indicates quotes from interviews with cPbL groups and “I” quotes from bPbL groups.

### Students’ statements in support of blended PbL

The categories relating to advantages of bPbL that were most frequently touched upon overall, but also by cPbL and bPbL groups, respectively, were the *low effort with regard to travel* and the *free choice of location*.

- C3, 57–59: “This is perhaps the advantage for people who now have a longer journey to get here”
- I8, 8-9: “One advantage was that you could stay at home and not drive to university for three quarters of an hour”
- C3, 47–48: “You just have to be able to be online somehow and then you can also do it from home”
- I5, 8: “That one can stay at home, i.e. [it is] independent of location”

Some statements supporting the blended PbL concerned the *atmosphere of the meeting*.

- C3, 138: “it’s just more comfortable”
- I4, 8: “During the event you could eat and drink and so on”

Some students said that participating via chat allowed them to *focus better* on what was important. Of note, such comments only came from students who had participated in the bPbL themselves.

- I7, 78–79: “Because I found just because you have to type it, you think about what you write”

Similarly, only students who had participated in bPbL themselves made positive comments with regard to technical aspects.

- I3, 70–71: “I’m not that tech-savvy myself, but I had no problems with it at all and I liked that”

### bPbL-sceptical statements by students

The category to which by far the most bPbL-sceptical statements could be assigned was “*Communication: implementation*”. Compared to cPbL groups, almost twice as many statements from bPbL groups referred to this category (*n* = 15 vs. *n* = 27).

- C8, 30–31: “Exactly, this answering one after the other, that probably doesn’t work in the chat”
- C9, 14-16: “I always have the feeling, like for example in a group chat now on WhatsApp, that it’s always so confused”
- I4, 42–43: “But now also just with the discussion, that’s just difficult somehow”
- I4, 99-102: “Simply because you were reading, then making your own thoughts, then typing, then scrolling up again to see if anyone had already written that, only to realise, oh crap, someone has already written that, I’ll delete it now, rewrite it again”

Related to this, one cPbL student suspected that there would often be *duplication* of content in the chat. Indeed, several statements (*n* = 10) by students from bPbL groups confirm that this actually happened.

- C5, 33–36: “then maybe someone else has already written this and then you have just written this”
- I6, 131-133: “Because that was really the case yesterday, that there was really double and triple any content, i.e. the same content”

With respect to *technical aspects* there were clearly more statements sceptical about than supporting bPbL.

- C6, 66: “Technical problems, if something is not working”

Some comments on technical aspects also had a socio-economic facet.

- C9, 53-55: “There are also disadvantages regarding the person who may not have the financial means to equip themselves technically to be able to participate well”
- I8, 190–191: “Is it even possible to assume that everyone has a laptop?”

Time saving was a frequent argument in favour of the bPbL (see above), whereby this mainly referred to the elimination of the need to travel. On the other hand, there were fears and experiences that communicating by writing in the chat is associated with *time loss*.

- C3, 19-20: “No, I would be totally lost there, because I just need so long to write until I’ve said my opinion, they’re already on to the next topic”
- I5, 35–36: “which then takes ages until you have actually typed in your message and got it across”

Both cPbL and bPbL students often expressed scepticism about the *learning effect*.

- C9, 23-26: “And I also have the impression that you learn that better [on site]. So you often remember things that you have discussed somewhere and then maybe there was some anecdote about it and I can personally remember that better”
- I5, 41-42: “That you pick up more and are more concentrated when you sit together in the room here than when you sit at home in front of your PC and just read along as a chat”

Many comments referred to the (lack of) *personal contact*. Although more distinguishable statements could be derived in total from the interviews with bPbL groups, concerns referring to this category came twice as often from cPbL students.

- C4, 91-93: “I think we all agreed that we would also like to have this social aspect, that we should also meet in this PbL”
- I7, 193-196: “So I also have the feeling that I would learn more if I somehow, simply connected the spoken word with a person, somehow just this interaction and the personal contact with them”

Similarly, concerns that students’ *active participation* would be compromised in the chat were more common among cPbL students.

- C4, 10–11: “Many then probably also simply hold back with their answers or simply confirm”
- I9, 29-30: “One is then partly inhibited to write that in there, because one is perhaps not so sure after all”

### Statements exclusively from either bPbL or cPbL students

There have been categories referring exclusively to statements from either bPbL or cPbL groups. With the exception of supportive statements from bPbL students regarding *technical simplicity* (*n* = 6) or *increased concentration* (*n* = 4), these statements were sporadic.

One bPbL student had concerns about *data protection*.

- I4, 30-32: “Because maybe I don’t want everything I said somewhere to be recorded and everyone else to be able to see it for a long time”

Another bPbL student saw the computer-based approach as a good *preparation for future work*.

- I9, 19–20: “I also thought it was good because it might also introduce us to our future work”

Two cPbL students emphasised the advantage of *eye contact* at the on-site meeting.

- C9, 12-23: “So I think it’s super important simply to have eye contact with the group, so that you can see each other”

One student—again from a cPbL group—referred to the fact that *body language* can be perceived on site.

- C6, 18–19: “You also get all the facial expressions and gestures from the others”

Two other cPbL students pointed out that on site, but not in the chat, there is the possibility of *visualisation*.

- C5, 67-68: “Yes, also has the advantage when you meet is that you can then record something like on a whiteboard or so”

## Discussion

Shortly before the COVID-19 pandemic outbreak, we investigated whether the first of two PbL meetings could be realised by means of blended learning, i.e. in our study as an internet chat instead of on-site. We found that this bPbL approach was feasible and did not differ from cPbL in terms of student evaluation, additional learning time and exam results. However, there was an effect on attending lectures on site or watching lecture videos. As expected, group interviews revealed that advantages of blended PbL are mainly seen in the free choice of location and time savings. However, there are also interesting differences between the assessments by bPbL and cPbL students.

According to Verstegen et al., e-learning approaches in PbL can be used to support contextual learning and/or collaborative learning (Verstegen et al. 2016). The first objective is predominantly attempted with measures that could be described as “digital enrichment”. It is important to emphasise that we did not pursue this goal in our study. Thus, our study mainly aimed at collaborative learning, but in the broader sense of a non-inferiority trial. We saw no differences between bPbL and cPbL groups with regard to satisfaction with the course, a scale that included items on tutor behaviour, group engagement with the subject and perceived preparation for the exam. Furthermore, results in the final summative exam did not differ between students having attended either bPbL or cPbL. Findings from an own previous study suggested that students tried to compensate for perceived (perhaps putative) deficits in the PbL process by increasing their learning time before the exam (Matthes et al. 2002). However, in line with the similar results in the written exam and the evaluation, we found no differences in the additional learning time in the current study. Taken together, our results suggest that our blended PbL approach was similar in terms of learning process, learning success and student acceptance. This may be surprising given the rather simple (“low fidelity”) approach we had chosen. In this context, it should be noted that attitudes towards CbL also did not differ between bPbL and cPbL students at the end of the course. This is not to be taken for granted, considering that even a sophisticated, well-designed programme can worsen students’ positive attitudes towards CbL (Hahne et al. 2005). At this point, it should be pointed out again that the main intervention we looked at in our study was conducting the first PbL session as an Internet chat instead of an on-site meeting, i.e. only the communication channel was different. The only (digital) addition compared to the conventional approach was the possibility to exchange ideas in an internet forum between the first and second meeting. With our approach we thus were in line with the suggestions made by Howard Barrows regarding “distributed PBL” (Barrows 2002): We combined synchronous and asynchronous electronic communication, but without interfering with the established PbL process. In fact, the above-mentioned internet forum was not used at all. We did not ask for reasons in the evaluation. However, students would have had to log in to use the forum, which was probably just too inconvenient in times when immediate communication in forums via messenger services like WhatsApp is taken for granted.

Compared to the cPbL students, relatively more students in the bPbL groups stated that they had not attended a single lecture on site. This was not necessarily to be expected, as the second PbL meeting, which was held on site for everyone, was immediately preceded by a lecture as part of the course, and events from other courses were also held on site. It is also interesting to note that participants in the conventional PbL were less likely to watch lecture videos, although the advantages were the same (e.g. timing at will, interruption possible at any time, repeated viewing possible). It is tempting to speculate that it was the blended PbL approach that first drew students’ attention to the benefits of further e-learning offers.

The results from the group interviews show that limitations in communicating and the lack of personal contact are seen as major shortcomings of our blended PbL approach. Furthermore, there were fears that the learning effect would be lower. However, this disadvantage does not seem to be serious, as shown by the non-different exam results on the one hand and the comparable satisfaction with the course on the other. Although the frequency of statements made in interviews should be interpreted cautiously, it is noticeable that students who had not participated in blended PbL themselves expressed concerns more frequently with regard to the lack of personal contact. That bPbL students raised this concern less often indicates that this disadvantage, too, was actually rather not that serious. On the other hand, bPbL students obviously experienced drawbacks that cPbL students tended not to be concerned about. For example, some bPbL students found communication via chat rather time-consuming and pointed out more often that statements in chat can be repetitive. In summary, our interview data largely confirms the evaluation data. Mostly similar advantages and disadvantages were expected or observed from cPbL or bPbL students, which fits with the similar attitude towards CbL in the evaluation. The fact that students who participated in blended PbL did not describe any serious disadvantages in the interviews fits with the similar course satisfaction in the evaluation by cPbL and bPbL students.

Using e-learning approaches for PbL is not new (Bridges et al. 2016). Although this can be seen as a weakness of a current study, it is rather a strength for two reasons: Firstly, technology is developing rapidly and so are the demands on the part of students. In this respect, it is interesting to see whether and how e-learning approaches are accepted nowadays. Since many technical possibilities (e.g. video telephony via smartphones) have become a matter of course, the question arises as to how “low fidelity” approaches (such as communication via chat in our study) are received. Secondly, the pandemic has led (at least temporarily) to a massive push in the field of digital teaching. Since our study was conducted shortly before the pandemic, it provides data in a context that may be more comparable than data collected 10 years ago or more. It can be assumed that, against the background of the experiences made during the pandemic, blended-learning options will increasingly become standard in teaching on the one hand, while on the other hand personal contacts will be appreciated all the more (Eringfeld 2021; McGrath et al. 2021). Our results indicate that in the case of PbL, chat can be an appropriate option at least for the first of two group meetings.

### Limitations

There are some limitations of our study to be considered. As mentioned above, the fact that we conducted our study before the COVID-19 pandemic can be seen as both a disadvantage and an advantage. Although our intervention aimed at collaborative, not contextual learning, we did not analyse the PbL process itself, tutor behaviour, or the learning goals derived from group discussions. This should be addressed in future studies. One should be very careful with interpreting findings from qualitative analyses in a (semi-) quantitative manner. Therefore, we have of course only described these results and not analysed them statistically. The results of a single-choice exam only partially reflect the learning success in PbL (Davis and Harden 1999; Azer 2003). Furthermore, studies suggested an inferiority of PbL with regard to results in those exams (Strobel and van Barneveld 2009), although more recent meta-analyses indicate rather similar or even enhanced knowledge scores (Zahid et al. 2016; Wang et al. 2016; Trullàs et al. 2022). It should be mentioned that we chose the exam format not with a study endpoint in mind, but it has been the standard examination instrument in this course for years.

### Conclusion

Combining on-site teaching and digital approaches in terms of blended learning has already been used in PbL. However, not much is known about simple (“low fidelity”) approaches in general and nowadays in particular. The COVID-19 pandemic necessitated a shift to digital learning formats, making it all the more important to think about approaches that are both feasible and sufficient in the post-pandemic period. Overall, our blended PbL has proven to be accepted while it did not affect exam results. Thus, we conclude that it could be a hybrid approach to digital teaching even in the post-pandemic era.

## Data Availability

The datasets generated during and/or analysed during the current study are available from the corresponding author on reasonable request.
